# Association between Heavy Metal Exposure and Dyslipidemia among Korean Adults: From the Korean National Environmental Health Survey, 2015–2017

**DOI:** 10.3390/ijerph19063181

**Published:** 2022-03-08

**Authors:** Do-won Kim, Jeongwon Ock, Kyong-Whan Moon, Choong-Hee Park

**Affiliations:** 1 Environmental Health Research Division, National Institute of Environmental Research, Ministry of Environment, Incheon 22689, Korea; done35005@gmail.com (D.-w.K.); ockjeongwon@korea.kr (J.O.); 2 BK21 FOUR R & E Center for Learning Health System, Department of Health and Environmental Science, Korea University, Seoul 02841, Korea

**Keywords:** heavy metals, serum lipid profiles, dyslipidemia, KoNEHS

## Abstract

Cardiovascular disease (CVD) is a leading cause of death in Korea. Dyslipidemia, characterized by the presence of abnormal lipid levels, has been suggested as an early diagnostic and preventable factor for CVD. Recent studies have shown that exposure to lead (Pb), cadmium (Cd), and mercury (Hg) affects lipid metabolism. This study aimed to verify the association between heavy metal concentrations and serum lipid profiles in the general population. A representative sample of 2591 Korean adults from the Korean National Environmental Health Survey (2015–2017) was analyzed. The associations between heavy metals [Blood Pb (BPb), blood Hg (BHg), urinary Hg (UHg), urinary Cd (UCd)] and serum lipid profiles [total cholesterol (TC), triglyceride (TG), low-density lipoprotein cholesterol (LDL-C), non-low level of high-density lipoprotein cholesterol (Non-HDL-C)] were assessed using regression analysis. After adjusting for demographic and socioeconomic factors, the proportional changes in serum lipid levels were significantly associated with increases in BPb, BHg, and UHg levels (*p* for trend < 0.05). Overall, BPb, BHg, and Uhg levels positively correlated with dyslipidemia, whereas UCd levels did not show a significant association. Our results suggest that heavy metal exposure, at low levels, may contribute to an increased prevalence of dyslipidemia in Korean adults.

## 1. Introduction

Hypertension, hyperglycemia, and dyslipidemia are observed in individuals with metabolic syndrome [1]. Dyslipidemia is an important risk factor of cardiovascular disease (CVD) which is one of the diseases with high mortality worldwide [2]. In particular, dyslipidemia causes lipid accumulation in the arterial wall, which reduces blood flow to the heart, leading to CVD [3,4,5]. The prevalence of dyslipidemia is 53% and 45% in the United States and Canada [2,6] respectively. In Korea, the prevalence has increased from 34.1% in 2010–2013 to 36.5% in 2013–2015 [7,8]. Dyslipidemia is characterized by an imbalance of lipid levels in the blood, and it is caused by excessive entry of lipoproteins into the bloodstream or impaired ability to remove them [9,10,11].

Dyslipidemia is generally defined as elevated total cholesterol (TC), triglyceride (TG), low-density lipoprotein cholesterol (LDL-C), and a low level of non-high-density lipoprotein cholesterol (Non-HDL-C) [12,13,14]. Lifestyle features, such as dietary habits, lack of exercise, and alcohol consumption are commonly known risk factors for dyslipidemia [15]; however, several recent epidemiological studies suggest that abnormal lipid metabolism is associated with environmental chemical exposure [16,17,18,19]. Heavy metals, such as lead (Pb), cadmium (Cd), and mercury (Hg), are released into the environment by natural and anthropogenic sources [20,21]. Human anthropogenic activities (e.g., agricultural, household, and industrial activities) have increased exposure to heavy metals through respiratory and dietary intake [22]. Environmental exposure to heavy metals occurs through breathing contaminated air, eating plants, and drinking water from contaminated soil or groundwater [23]. 

Heavy metals can accumulate in various tissues and exhibit toxic effects on intracellular metabolic processes (DNA damage, oxidative proteolysis, etc.) [24]. As a result, heavy metal exposure can cause various diseases [25]. Oxidative stress is one of the most important consequences of heavy metal exposure [26]. In particular, the production of hydroxyl (•OH) and peroxynitrite (ONOO-), which are involved in oxidative stress, are known to cause lipid imbalance and CVD [27,28]. In conclusion, heavy metal exposure may affect an increase in cholesterol, triglyceride, and lipoprotein content [28]. According to previous studies, Xu et al. (2021) reported significant increases in TC, Non-HDL-C, and LDL-C levels with Pb exposure in the US adult population [29]. A significant association has been proposed between Cd-exposed Korean adults and an increased risk of dyslipidemia [30]. Participants with above-average Hg concentrations were reported to have a higher risk of dyslipidemia than those who did not [31]. However, heavy metal toxicity depends on several factors, including the age, gender, genetics, and nutritional status of the exposed individual [32]. As heavy metal concentrations in Korean are higher than those in developed countries [33], low levels of chronic heavy metal exposure remain a public health concern. In addition, only a few studies have been conducted to investigate the association between heavy metal exposure and dyslipidemia in Koreans. Therefore, it is necessary to confirm the effect of heavy metals on the serum lipid profile of a representative adult sample of Korea. In the present study, we investigated blood and urine heavy metals (Pb, Cd, Hg), as well as serum lipid profiles (0, TG, LDL-C, non-HDL-C) provided in the Korea National Environmental Health Survey (KoNEHS) 2015–2017.

## 2. Methods 

### 2.1. Study Population

**Figure 1:**
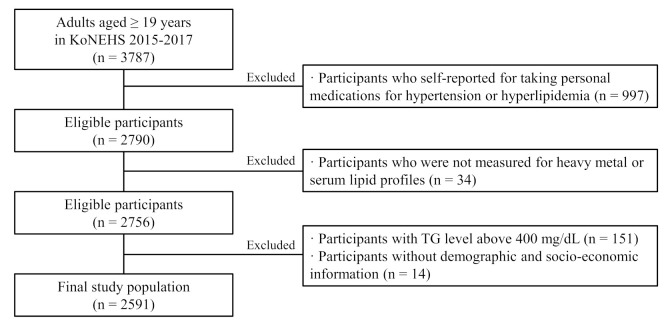
Participant flow chart of Korea National Environmental Health Survey (KoNEHS) 2015–2017 data available in this study.

The KoNEHS is a cross-sectional survey conducted every 3 years since 2009. The survey was designed to estimate and monitor exposure to various environmental pollutants in a representative Korean adult population. We analyzed the association between heavy metal exposure and serum lipid profile using data from 2015 to 2017, provided by KoNEHS. The national survey consists of a questionnaire, a physical examination, and the collection and analysis of biological samples, including a total of 26 chemicals and 16 clinical trial samples. Through a survey of trained investigators, six categories of information were obtained, including demographic and socioeconomic questions. Biological samples were collected by medical technicians and nurses under the supervision of a physician. In KoNEHS cycle 3, urine and blood samples were collected from a total of 3787 adults, aged 19 years and older. The following participants were excluded from the study (Figure 1): those who self-reported taking medications for hypertension or hyperlipidemia (*n* = 997), those who were not tested for heavy metals or serum lipid profiles (*n* = 34), and those with TG levels above 400 mg/dL (*n* = 151), according to the Friedewald’s equation, used to calculate LDL-C levels. Participants without urine creatinine concentration information, as well as demographic and socio-economic information used as covariates (*n* = 14), were also excluded. Therefore, a total of 2591 participants were used for the analysis. This study was approved by the Research Ethics Committee of the National institute of Environmental Research [34].

### 2.2. Measurement of Heavy Metals

KoNEHS analyzed three heavy metals, including Pb, Cd, and Hg. Among these, Pb and total Hg were analyzed in blood, while Hg and Cd were analyzed in spot urine. Blood samples and spot urine samples were collected using a blood collection vessel (VACCUTAINER Royal blue cap) and a sterile urine collection vessel (sample cup), respectively. The samples were maintained at 2–6 °C, using an ice box, and refrigerated at −20 °C for future analysis. Blood Pb (BPb) and urine Cd (UCd) were analyzed using graphite furnace-atomic absorption spectroscopy (AAnalyst-800, Perkin Elmer) at wavelengths of 283.3 and 228.8 nm, respectively. Mercury concentrations in blood (BHg) and urine (UHg) were analyzed at wavelengths of 254.65 and 253.7 nm, respectively, using a gold amalgamation direct mercury analyzer (DMA-80, Milestone). The limits of detection (LOD) of heavy metals (Pb, Cd, and Hg) were 0.3 μg/dL, 0.1 μg/L, and 0.05 μg/L, respectively [35]. Values below the LOD of Pb, Cd, and Hg were substituted with the square root of the LOD. The quality control procedure was conducted using the standardized protocol procedure of the National Academy of Environmental Sciences. 

### 2.3. Measurement of Serum Lipid Profile

KoNEHS collected total cholesterol (TC), Triglyceride (TG), and high-density lipoprotein cholesterol (HDL) from serum samples. Whole blood samples were collected in serum separation tubes (SST). The samples were mixed by inversion, held for 30 min, and then, centrifuged at 3500 RPM (revolutions per minute). TC, TG, and HDL-C levels were analyzed using an automated analyzer (ADVIA 1800; Siemens Medical Solutions, Ann Arbor, MI, USA) at 505/694, 505/694, and 596 nm wavelengths, respectively. The limits of detection (LOD) for TC, HDL-C, and TG were 10.0, 5.0, 10.0 mg/dL, respectively [36]. Values below the LOD of TC, HDL-C, and TG were replaced with the square root of the LOD.

### 2.4. Evaluation of Serum Lipid Profile

Dyslipidemia was defined as the presence of one or more of the following: TC ≥ 200 mg/dL, TG ≥ 150 mg/dL, LDL-C ≥ 130 mg/dL, and non-HDL-C ≥ 160 mg/dL [12,37]. In this study, LDL-C and non-HDL-C levels were obtained using indirect methods. LDL-C was calculated as TC−HDL(C)−[TG/5] using Friedewald’s formula [38]. Owing to a limitation of the formula, TG values greater than 400 mg/dL were treated as missing values [39,40]. This is because TG levels above 400 mg/dL may be underestimated, owing to a decrease in the TC:TG ratio of very low-density lipoprotein cholesterol (VLDL-C) [38]. Non-HDL-C was calculated as TC − HDL(C) [41]. Indirectly estimated non-HDL-C levels may be a better indicator of atherosclerotic lipoprotein particles than HDL-C levels [42,43]. KoNEHS samples could be used since fasting conditions are not required for lipid analysis.

### 2.5. Covariates

Covariates were chosen based on the associations between serum lipid profiles, according to the following characteristics provided in previous epidemiological studies [22,44]: age (19–39, 40–59, and 60 years or older); sex (Male, female); body mass index (BMI) calculated by dividing body weight (kg) by the square of height, and then, divided into categories of underweight (<18.5), normal (18.5–23), overweight (23–25), and obese (>25), according to the criteria of the Korean Society for the Study of Obesity (KOSSO); household income [low < 840; medium, 840–4220; high ≥ 4220 US dollars]; education (≤middle school; high school; ≥college); smoking status (non-smoker, former smoker, current smoker); alcohol consumption [non-drinkers, light drinkers (twice a month), heavy drinker (once a week to almost daily)]; physical activity (none, moderate, and rarely).

### 2.6. Statistical Analysis

Stratum and cluster weights were applied to the regression model to consider a two-step proportional stratified sample design for KoNEHS data. For the analysis, differential probabilities of selection, non-response, and sample weights were applied. Serum lipid profiles were normally distributed and calculated as the arithmetic mean. The heavy metal concentrations did not follow a normal distribution, and the geometric means was calculated by log-transformation. Student’s *t*-test and analysis of variance (ANOVA) were used to analyze statistical differences between different groups for continuous variables. Heavy metal concentrations were divided into quartiles, and serum lipid profiles were used as continuous variables. The mean change in serum lipid profile concentration by heavy metal concentration was analyzed using multiple linear regression analysis. For multivariate logistic regression, serum lipid profiles were divided into two groups, based on cholesterol levels (dyslipidemia or non-dyslipidemia by cholesterol levels) and the odds ratio [OR, 95% confidence interval (CI)] of dyslipidemia by heavy metal concentration. A value of *p* < 0.05 was considered as statistically significant in all analyses. Statistical analysis was performed using the Statistical Analysis System (SAS) 9.4 software.

### 2.7. Sensitivity Analysis

First, as a limitation of the use of the LDL-C estimation formula, exclusion of participants with TG ≥ 400, may have confounded the results of other serum lipid profiles. Therefore, we performed an additional sensitivity analysis, for OR, of serum lipid profiles (TC, TG, and non-HDL-C) by heavy metal concentration in 2742 participants, including TG ≥ 400. Second, after further adjustment for heavy metal-related occupations for high exposure, the association between heavy metal exposure and serum lipid profile was analyzed. This analysis was performed on 74 participants involved in occupations related to heavy metals among 2951 participants with occupational data. Third, since non-occupational Hg exposure is primarily due to seafood consumption, we further adjusted for the high seafood dietary group. The high seafood consumption group was calculated by the sum of the frequency of consuming one or more of the five items (large fish and tuna, crustaceans, shellfish, and other seafood) at least once a week. Therefore, 1627 participants were classified into the high seafood dietary group, and the remaining 964 participants constituted the other group.

## 3. Results

### Demographic Characteristics and Serum Lipid Profiles

The weighted arithmetic means (AM) of TC, TG, LDL-C, and non-HDL-C were 185.70 (0.97), 145.42 (2.17), 99.10 (0.87), and 128.18 (0.97) mg/dL, respectively (Table 1). Serum lipid profile levels were significantly higher in male than in female, and they significantly increased with age and BMI (*p* < 0.001). In participants with low household income, TG significantly increased (*p* < 0.05). Participants with low education levels had higher serum lipid profile levels (*p* < 0.05). Participants who smoked had significantly higher lipid levels than those who did not (*p* < 0.05). TG was significantly linked with drinking status (*p* < 0.05). In terms of physical activity, only TG was significantly lower in the group, with hardly any physical activity compared with the reference group (*p* < 0.05).

Table 2 shows the comparison of heavy metal concentrations in the non-dyslipidemia and the dyslipidemia group. Concentrations of BPb in the elevated lipid profile group of TC, TG, LDL-C, and Non-HDL-C were significantly higher than that in the non-dyslipidemia group (*p* < 0.05). BHg concentrations were significantly higher in the dyslipidemia group of TC, TG, LDL-C, and Non-HDL-C than that in the non-dyslipidemia group (*p* < 0.05). UHg concentrations were significantly higher in the dyslipidemia group of LDL-C and Non-HDL-C than that in the non-dyslipidemia group (*p* < 0.05). The UCd concentrations were significantly higher in the dyslipidemia group of LDL-C and Non-HDL-C than that in the non-dyslipidemia group (*p* < 0.05).

Table 3 presents the adjusted ORs (95% CIs) for dyslipidemia by heavy metals. BPb and BHg concentrations showed significant associations with a higher risk of dyslipidemia (all *p* < 0.05). For BPb, the ORs of the upper quartiles of elevated TC, LDL-C, and non-HDL-C compared with the OR of the lowest quartile were 1.49 (95% CI: 1.07–2.08; *p* = 0.084), 1.57 (95% CI: 1.02–2.40; *p* = 0.041), 1.71 (95% CI: 1.09–2.68; *p* = 0.049), respectively. Compared with the OR in the lowest quartile of BHg, the OR of elevated TC in the 4th quartile was 1.72 (95% CI:1.21–2.44; *p* = 0.016), and ORs for elevated LDL-C for the 2nd, 3rd, and 4th quartiles, compared with the OR of the 1st quartile, were 1.63 (95% CI: 1.08–2.45), 1.77 (95% CI:1.17–2.68), and 2.21 (95% CI:1.49–3.28), respectively (*p* for trend < 0.05). There are no significant associations between Uhg, UCd, and dyslipidemia. Additionally, we examined the association between heavy metals and serum lipid profiles for including TG ≥ 400. The results were similar to those obtained, with the exclusion of TG ≥ 400 (Supplementary Materials, Table S1). After adjustment for heavy metal-related occupations or the high seafood dietary group, the results were consistent with those obtained with the non-adjustment models (Supplementary Materials, Tables S2 and S3).

**Figure 2:**
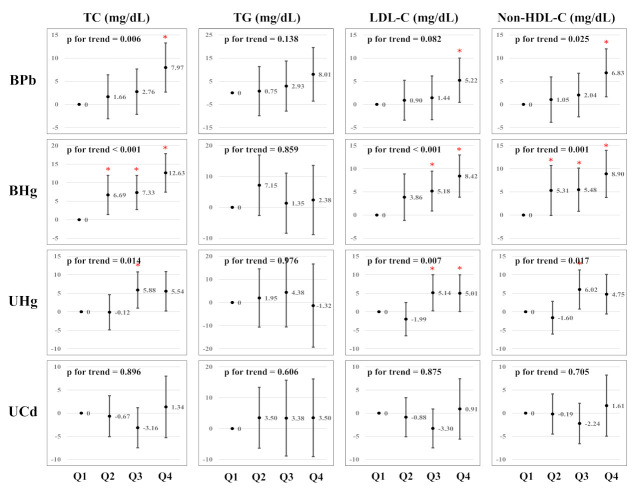
Multiple linear regression analysis of serum lipid profile by heavy metal concentrations (*n* = 2591). Asterisks (*) indicate significance level (<0.05).

Figure 2 presents the estimated associations between serum lipid profiles and heavy metal concentrations using multiple linear regression analysis. After multivariable adjustment, BPb, BHg, and UHg in the upper quartiles had higher TC, TG, LDL-C, and non-HDL-C levels than those in the lowest quartile. There is evidence of dose-response relationships between: BPb and TC, LDL-C, and non-HDL-C; BHg and TC and Non-HDL-C; UHg and TC, LDL-C, and Non-HDL-C (*p* for trend <0.05). We did not observe dose–response relationships between the UCd concentrations and serum lipid profiles.

## 4. Discussion

In a representative sample of Korean adults, using KoNEHS 2015–2017, we examined the association between heavy metal exposure and serum lipid profiles. After adjusting for all covariates in multivariate regression analysis, it was observed that there was a positive association between high BPb, BHg, Uhg, and serum lipid profiles, including TC, LDL-C, and non-HDL-C. In addition, high BPb and BHg were observed to be significantly associated with dyslipidemia in logistic regression analysis.

The Ministry of Environment has been involved in ongoing efforts to reduce environmental exposure to heavy metals since 1988 [45]. According to data from the KoNEHS (2009–2017), the concentration of heavy metals in Korean adults is decreasing, but it is still higher than that found in the adults of developed countries [33]. The Korean geography (island/coastal country) may influence high heavy metal concentrations, owing to the culture of high seafood consumption [39]. Seafood consumption has been reported as one of the main sources of heavy metal exposure in the general population [46]. In addition, soil and groundwater contamination, caused by abandoned mines in Korea, is a serious social problem [47]. The traditional Asian food culture, which frequently consumes grains and herbal medicines grown in contaminated soil, is supported by the high concentration of heavy metals in Korea [48].

Previous epidemiological studies have suggested an association between dyslipidemia and heavy metal exposure. According to a study using the National Health and Nutrition Examination Survey (NHANES), the ORs for TC, non-HDL-C, and LDL-C were 1.88, 1.59, and 1.68 times higher in the highest BPb quartile (>1.90 μg/dL) than that in the lowest quartile (≤0.76 μg/dL), respectively [29]. Another study found that TC and LDL-C levels were 1.5–2.0 and 1.6–2.4 times higher (*p* < 0.001) in the occupational Pb exposure group (27.00–48.90 μg/dL for BPb) than in the control group (15.78 μg/dL for BPb), whereas TG and HDL-C levels did not show a significant association (*p* < 0.05) [49]. Furthermore, a study using the KoNEHS observed that BHg concentrations are significantly higher in the hyperlipidemia group (4.03 and 2.83 μg/L for male and female) than that in the non-hyperlipidemia group (3.48 and 2.69 μg/L of male and female), and individuals with higher BHg concentrations have a 11% higher risk of hyperlipidemia [50]. Significant increases in TC, HDL-C, and LDL-C levels are reported with increasing BHg concentrations in Korean adults, based on the KoNEHS [40,51].

The toxic mechanism of lipid metabolism by heavy metal exposure is not yet completely understood, but it is generally associated with oxidative stress. Oxidative stress is caused by an imbalance between the antioxidant defense system and the free radical production system [24]. Heavy metal binds to sulfhydryl protein groups and depletes glutathione, leading to oxidative stress through reactive oxygen species (ROS) generation [52]. Oxidative stress activates several protein phosphorylation pathways, including phosphatidylinositol 3-kinase (PI3K) and MAPK, c-Jun N terminal kinase (JNK), and stress-activated protein kinase (SAPK) [53]. This activity is involved in the apoptotic process of pancreatic β-cells responsible for insulin production, which plays a major role in glycemic homeostasis, leading to insulin resistance [20,54]. This condition can lead to endothelial dysfunction through decreased vasodilator production and increased vasoconstrictor production [55]. Animal studies have demonstrated that heavy metal exposure impairs glucose metabolism by pancreatic β-cell damage. In vitro and in vivo studies have proved that chronic exposure to Pb affects the pancreas and disrupts glucose homeostasis [56,57]. Exposure to Hg induces ROS production, impaired insulin secretion, and apoptosis of β-cell-derived HIT-T15 cells [58,59].

In our study, UCd concentrations were not significantly associated with elevated serum lipid levels. According to a previous study, a positive correlation was observed between BCd concentrations and HDL-C risk in Korean adults [30]. In addition, an increased prevalence of high TC, TG, and LDL-C, as well as low HDL-C, with increasing BCd concentrations was observed in Chinese occupationally exposed to Cd [60]. In contrast, several studies using NHANES data have shown no significant association between UCd or BCd levels and metabolic syndrome risk [61,62], which is consistent with our study findings. These conflicting results might be due to differences in individual participant characteristics, exposure levels, sampling bias, size, and covariate settings [29]. Some, kinds of biosamples (i.e., urine or blood) might affect the associations because UCd serves as a biomarker of long-term cadmium exposure, and BCd serves as a biomarker of recent cadmium exposure, [63,64].

Our study has several limitations that warrant discussion. First, it is a cross-sectional observational study based on a single measurement. Thus, it cannot indicate the exact causal relationship of the observed association. Second, since heavy metals in blood reflect relatively recent exposure, there is a limit to explaining the burden on the body due to long-term exposure. Third, although this study focused on Pb, Cd, and Hg exposure, these may be accompanied by potential exposure to other metals.

However, our study has several notable advantages. First, we used individual blood or urine heavy metal concentrations to account for heavy metal exposure levels, and we observed the association with serum lipid profiles. Second, we used three types (Pb, Cd, and Hg) of heavy metal biomarkers to provide the opportunity to observe different associations between each heavy metal exposure and serum lipid profiles. Third, our results can be interpreted as the results of the national population because we used data from KoNEHS, a large representative sample of Korean adults. The results of our study provide epidemiological evidence for an association with dyslipidemia and current heavy metal exposure levels in Koreans.

## 5. Conclusions

We assessed the association between heavy metal exposure and dyslipidemia using data from a nationally representative adult population in Korea. Our findings showed a positive association between BPb, BHg, and UHg concentrations and serum lipid profiles. In addition, BPb and BHg concentrations were positively associated with a prevalence of dyslipidemia. These results support evidence that heavy metal exposure may be an important risk factor for abnormal lipid metabolism. Results from this study, in conjunction with others, strengthen the concern on heavy metal effects at low-level when evaluating heavy metal regulations concerning adverse health effects. However, further studies are needed to elucidate the mechanisms supporting the association between heavy metal exposure and dyslipidemia in the general population.

## Figures and Tables

**Table 1:** Arithmetic means (mg/dL) of serum lipid profiles (mg/dL) in participants (*n* = 2591) by demographic characteristics.

	*n* (%)		TC (mg/dL (SE))		TG (mg/dL (SE))		LDL-C (mg/dL (SE))		Non-HDL-C (mg/dL (SE))	
**Total**	2591	(100)	185.70	(0.97)	145.42	(2.17)	99.10	(0.87)	185.70	(0.97)
**Sex**										
Male	1111	(49.4)	182.52	(1.67)	145.45	(3.08)	95.00	(1.51)	128.48	(1.63)
Female	1480	(50.6)	182.58	(1.11)	110.71	(1.98)	93.86	(0.97)	119.03	(1.12)
*p*-value			<0.001		<0.001		0.249		<0.001	
**Age group (years)**										
19–39	766	(29.56)	175.58	(1.54)	116.95	(3.11)	88.32	(1.41)	115.55	(1.51)
40–59	1152	(44.46)	190.12	(1.20)	134.35	(2.34)	100.57	(0.99)	131.07	(1.14)
≥60	673	(25.97)	183.13	(2.06)	137.16	(3.53)	96.81	(1.83)	128.63	(2.07)
*p*-value			<0.001		<0.001		<0.001		<0.001	
**BMI (kg/m^2^)**										
<18.5	82	(3.92)	174.05	(5.25)	95.39	(6.33)	89.15	(3.62)	108.23	(4.01)
18.5–22.9	944	(39.0)	177.41	(1.33)	115.01	(2.82)	91.84	(1.11)	114.84	(1.25)
23.0–25.0	677	(25.9)	191.55	(1.95)	154.45	(3.47)	104.59	(1.92)	135.48	(2.03)
>25	888	(31.8)	192.36	(1.75)	180.91	(4.00)	104.61	(1.56)	140.80	(1.64)
*p*-value			<0.001		<0.001		<0.001		<0.001	
**Household income (US$/month)**										
Low (840)	332	(12.81)	180.64	(3.08)	139.78	(5.73)	94.08	(2.75)	126.74	(2.97)
Middle (840–4220)	1777	(68.58)	181.64	(1.09)	124.58	(2.23)	94.05	(1.01)	122.59	(1.08)
High (≥4220)	482	(18.60)	185.86	(2.02)	129.04	(4.42)	95.64	(1.78)	125.65	(1.98)
*p*-value			0.063		0.006		0.729		0.103	
**Education levels**										
≤Middle school	641	(24.74)	187.29	(1.69)	135.53	(2.99)	99.84	(1.60)	131.36	(1.60)
High school	847	(32.69)	184.23	(1.47)	130.27	(3.16)	96.52	(1.19)	126.35	(1.40)
≥College	1103	(42.57)	180.52	(1.37)	122.76	(2.66)	92.05	(1.31)	120.35	(1.32)
*p*-value			0.001		0.025		<0.001		<0.001	
**Smoking status**										
Never	1694	(65.38)	181.05	(1.05)	116.05	(2.03)	93.44	(0.92)	120.01	(1.06)
Former	455	(17.56)	187.55	(2.11)	149.54	(4.36)	99.08	(1.81)	133.42	(1.93)
Current	442	(17.06)	183.27	(2.15)	145.87	(5.08)	93.81	(1.88)	127.41	(2.21)
*p*-value			0.002		<0.001		0.001		<0.001	
**Drinking status**										
Never	783	(30.22)	182.08	(1.57)	123.99	(4.23)	95.32	(1.44)	123.86	(1.69)
Light	889	(34.31)	180.61	(1.78)	118.92	(3.07)	95.37	(1.48)	122.64	(1.77)
Heavy	919	(35.47)	184.57	(1.42)	135.69	(3.51)	93.06	(1.40)	124.31	(1.40)
*p*-value			0.118		<0.001		0.099		0.760	
**Physical activity**										
None	1458	(56.27)	182.55	(1.21)	126.51	(2.32)	94.40	(1.11)	123.51	(1.27)
Moderate	204	(7.87)	184.92	(3.00)	138.31	(7.15)	93.85	(2.86)	125.82	(3.57)
Hardly	929	(35.85)	182.08	(1.70)	124.70	(3.00)	94.58	(1.48)	123.31	(1.65)
*p*-value			0.631		0.014		0.856		0.575	

*p*-values are calculated based on survey *t*-test for binominal groups (sex) and based on Wald F-test for categorical groups (age groups, BMI, household income, education levels, smoking status, drinking status, and physical activity); Abbreviations: SE, standard error; TC, total cholesterol; TG, triglyceride; LDL, low-density lipoprotein cholesterol; HDL-C, high density lipoprotein cholesterol; Non-HDL, non-high density lipoprotein cholesterol.

**Table 2:** Geometric means of heavy metal concentrations, according to the dyslipidemia or non-dyslipidemia group (*n* = 2591).

	*n*	BPb (μg/dL (95% CIs))		BHg (μg/L (95% CIs)) ^a^		UHg (μg/L (95% CIs)) ^a^		UCd (μg/L (95% CIs))	
**TC (mg/dL)**									
**<200**	1703	1.50	(1.46, 1.55)	2.56	(2.41, 2.71)	0.38	(0.36, 0.41)	0.39	(0.36, 0.42)
**≥** **200**	888	1.64	(1.57, 1.71) *	3.06	(2.89, 3.24) *	0.42	(0.39, 0.45)	0.43	(0.39, 0.47)
**TG (mg/dL)**									
**<150**	2254	1.45	(1.40, 1.50)	2.53	(2.38, 2.68)	0.39	(0.36, 0.41)	0.38	(0.35, 0.42)
**≥** **150**	337	1.72	(1.65, 1.79) *	3.02	(2.85, 3.20) *	0.40	(0.38, 0.43)	0.43	(0.39, 0.47)
**LDL-C (mg/dL)**									
**<160**	2168	1.52	(1.47, 1.56)	2.62	(2.48, 2.76)	0.38	(0.36, 0.40)	0.39	(0.36, 0.42)
**≥** **160**	423	1.72	(1.63, 1.83) *	3.32	(3.10, 3.56) *	0.46	(0.41, 0.51) *	0.48	(0.43, 0.53) *
**Non-HDL-C (mg/dL)**									
**<130**	2091	1.51	(1.46, 1.55)	2.61	(2.48, 2.76)	0.38	(0.36, 0.40)	0.39	(0.36, 0.42)
**≥** **130**	500	1.76	(1.67, 1.86) *	3.20	(2.97, 3.45) *	0.45	(0.41, 0.50) *	0.45	(0.40, 0.50) *

^a^ Total mercury, * *p*-value < 0.05, Abbreviations: CI, confidence interval; BPb, blood lead; BHg, blood mercury; UHg, urinary mercury; UCd, urinary cadmium; TC, total cholesterol; Non-HDL, non-high density lipoprotein cholesterol; TG, triglyceride; LDL, low-density lipoprotein cholesterol.

**Table 3:** Multivariate logistic regression of serum lipid profiles by heavy metal concentrations in participants (*n* = 2591).

β (95% CI) of Serum Lipid Levels										
		*n*	Elevated TC (≥200 mg/dL)		Elevated TG (≥150 mg/dL)		Elevated LDL-C (≥130 mg/dL)		Elevated Non-HDL-C (≥160 mg/dL)	
**BPb (μg/dL)**										
**Q1**	(0.37–1.21)	651		Reference		Reference		Reference		Reference
**Q2**	(1.21–1.65)	644	1.05	(0.76, 1.45)	1.11	(0.77, 1.62)	0.96	(0.59, 1.53)	1.03	(0.66, 1.60)
**Q3**	(1.65–2.23)	647	1.14	(0.87, 1.50)	1.13	(0.78, 1.65)	1.24	(0.84, 1.84)	1.27	(0.84, 1.92)
**Q4**	(2.23–20.58)	649	1.49	(1.07, 2.08) *	1.43	(0.98, 2.08)	1.57	(1.02, 2.40) *	1.71	(1.09, 2.68) *
*p* for trend			0.084		0.249		0.041		0.049	
**BHg (μg/L) ^a^**										
**Q1**	(0.33–1.85)	647		Reference		Reference		Reference		Reference
**Q2**	(1.86–2.77)	648	1.17	(0.86, 1.59)	1.23	(0.81, 1.56)	1.63	(1.08, 2.45) *	1.20	(0.80, 1.79)
**Q3**	(2.77–4.30)	649	1.21	(0.87, 1.68)	1.00	(0.73, 1.37)	1.77	(1.17, 2.68) *	1.02	(0.64, 1.64)
**Q4**	(4.30–60.60)	647	1.72	(1.21, 2.44) *	0.86	(0.59, 1.24)	2.21	(1.49, 3.28) *	1.52	(1.01, 2.28) *
*p* for trend			0.016		0.478		0.012		0.011	
**UHg (μg/L) ^a^**										
**Q1**	(0.10–0.23)	655		Reference		Reference		Reference		Reference
**Q2**	(0.24–0.35)	641	0.87	(0.64, 1.18)	0.95	(0.66, 1.36)	0.89	(0.55, 1.44)	0.79	(0.51, 1.20)
**Q3**	(0.36–0.64)	648	1.19	(0.85, 1.66)	1.10	(0.74, 1.62)	1.36	(0.88, 2.11)	1.32	(0.86, 2.02)
**Q4**	(0.65–8.70)	647	1.32	(0.88, 1.96)	0.77	(0.52, 1.15)	1.55	(0.98, 2.45)	1.37	(0.89, 2.10)
*p* for trend			0.154		0.185		0.055		0.037	
**UCd (μg/L)**										
**Q1**	(0.05–0.22)	688		Reference		Reference		Reference		Reference
**Q2**	(0.22–0.45)	607	1.00	(0.70, 1.43)	1.18	(0.88, 1.58)	0.84	(0.58, 1.22)	1.16	(0.65, 2.06)
**Q3**	(0.45–0.87)	648	0.78	(0.56, 1.09)	1.06	(0.74, 1.52)	0.61	(0.39, 0.94)	1.72	(0.95, 3.11)
**Q4**	(0.87–16.82)	648	0.86	(0.59, 1.24)	1.14	(0.83, 1.56)	0.85	(0.56, 1.29)	1.58	(0.83, 2.99)
*p* for trend			0.396		0.657		0.661		0.172	

^a^ Total mercury, * *p*-value < 0.05, Abbreviations: CI, confidence interval; BPb, blood lead; BHg, blood mercury; UHg, urinary mercury; UCd, urinary cadmium; TC, total cholesterol; Non-HDL, non-high density lipoprotein cholesterol; TG, triglyceride; LDL, low-density lipoprotein cholesterol.

## Data Availability

This study used data from the Second Korean National Environmental Health Survey (KoNEHS) which was conducted by the Ministry of Environment, National Institute of Environmental Research. The data presented in this study are available on request from the corresponding authors. The data are not publicly available due to protect personal information.

## Supplementary Materials

The following supporting information can be downloaded at: https://www.mdpi.com/article/10.3390/ijerph19063181/s1, Table S1: Multivariate logistic regression of serum lipid profiles by heavy metal concentrations including TG ≥ 400 in participants (*n* = 2742); Table S2: High seafood dietary group-adjusted OR (95% CIs) of serum lipid profiles by heavy metal concentrations in participants (*n* = 2591); Table S3: Heavy metals related occupation-adjusted OR (95% CIs) of serum lipid profiles by heavy metal concentrations in participants (*n* = 2591).
