# Supplementary material for: Association between Heavy Metal Exposure and Dyslipidemia among Korean Adults: From the Korean National Environmental Health Survey, 2015–2017
Source: Int J Environ Res Public Health. 2022 Mar 8;19(6):3181. doi: 10.3390/ijerph19063181 (PMC8951064; doi:10.3390/ijerph19063181)
Supplement: Supplementary file 1 [file ijerph-19-03181-s001.zip › ijerph-1588804-supplementary.pdf]

Supplemental Material, Table S1. Multivariate logistic regression of serum lipid profiles by heavy metal concentrations including TG ≥ 400 in participants (n = 2,742)

	n	OR (95% CI) of serum lipid levels		
		**Elevated TC** (≥ 200mg/dL)	**Elevated TG** (≥ 150mg/dL)	**Elevated non-HDL-C** (≥ 160mg/dL)
**BPb (μg/dL)**				
**Q1** (0.37–1.22)	689	Reference	Reference	Reference
**Q2** (1.22–1.66)	682	1.05 (0.76, 1.43)	1.06 (0.75, 1.49)	1.02 (0.67, 1.55)
**Q3** (1.66–2.25)	687	1.15 (0.88, 1.51)	1.15 (0.80, 1.65)	1.30 (0.89, 1.92)
**Q4** (2.25–20.58)	684	1.40 (1.02, 1.93)*	1.35 (0.92, 1.96)	1.59 (1.04, 2.44)*
p for trend		0.03	0.109	0.01
**BHg (μg/L)[a]**				
**Q1** (0.33–1.86)	687	Reference	Reference	Reference
**Q2** (1.86–2.81)	684	1.15 (0.84, 1.57)	1.07 (0.78, 1.48)	1.14 (0.78, 1.65)
**Q3** (2.81–4.37)	686	1.14 (0.83, 1.55)	0.98 (0.71, 1.35)	0.91 (0.58, 1.42)
**Q4** (4.37–60.60)	685	1.61 (1.14, 2.26)*	0.85 (0.60, 2.21)	1.45 (1.98, 2.16)*
p for trend		0.01	0.365	0.132
**UHg (μg/L)[a]**				
**Q1** (0.10–0.23)	689	Reference	Reference	Reference

**Q2** (0.23–0.36)	679	0.83	(0.60, 1.13)	0.97	(0.69, 1.37)	0.82	(0.55, 1.21)
**Q3** (0.36–0.65)	691	1.06	(0.77, 1.45)	1.06	(0.73, 1.53)	1.17	(0.80, 1.73)
**Q4** (0.65–8.70)	683	1.19	(0.80, 1.76)	0.77	(0.53, 1.14)	1.26	(0.83, 1.91)
p for trend		0.284		0.324		0.136	
**UCd (μg/L)**							
**Q1** (0.05–0.22)	733	Reference		Reference		Reference	
**Q2** (0.22–0.45)	639	0.97	(0.68, 1.37)	1.09	(0.82, 1.46)	0.86	(0.61, 1.22)
**Q3** (0.45–0.86)	685	0.80	(0.57, 1.12)	1.10	(0.78, 1.55)	0.68	(0.44, 1.04)
**Q4** (0.86–16.81)	685	0.78	(0.54, 1.12)	1.03	(0.75, 1.40)	0.75	(0.50, 1.14)
p for trend		0.111		0.900		0.120	

[a]Total mercury

*p-value < 0.05

Abbreviations: CI, confidence interval, BPb, blood lead; BHg, blood mercury; UHg, urinary mercury; UCd, urinary cadmium; TC, total cholesterol; non-HDL, non-high density lipoprotein cholesterol; TG, triglyceride; LDL, low-density lipoprotein cholesterol

Covariates: age, gender, BMI, house income, education level, smoking states, drinking states, and physical activity

Supplemental Material, Table S2. High seafood dietary group-adjusted OR (95% CIs) of serum lipid profiles by heavy metal concentrations in participants (n = 2,591)

			β (95% CI) of serum lipid levels						
		**n**	**Elevated TC** (≥200 mg/dL)		**Elevated TG** (≥150 mg/dL)		**Elevated LDL-C** (≥130 mg/dL)		**Elevated-Non_HDL-C** (≥160mg/dL)	
**BPb (μg/dL)**									
**Q1**	(0.37–1.21)	651		Reference		Reference		Reference		Reference
**Q2**	(1.21–1.65)	644	1.05	(0.77, 1.45)	1.06	(0.65, 1.72)	0.95	(0.59, 1.52)	1.02	(0.65, 1.59)
**Q3**	(1.65–2.23)	647	1.14	(0.87, 1.50)	0.89	(0.55, 1.45)	1.24	(0.84, 1.84)	1.27	(0.84, 1.92)
**Q4**	(2.23–20.58)	649	1.49	(1.07, 2.07)*	1.05	(0.61, 1.80)	1.57	(1.03, 2.40)*	1.71	(1.09, 2.68)*
p for trend			0.02		0.943		0.02		0.01	
**BHg (μg/L)** [a]									
**Q1**	(0.33–1.85)	647		Reference		Reference		Reference		Reference
**Q2**	(1.86–2.77)	648	1.19	(0.87, 1.61)	1.29	(0.79, 2.09)	1.62	(1.07, 2.44)*	1.93	(1.10, 3.41)
**Q3**	(2.77–4.30)	649	1.24	(0.89, 1.72)	1.00	(0.62, 1.63)	1.76	(1.45, 3.29)*	1.56	(0.89, 2.76)
**Q4**	(4.30–60.60)	647	1.78	(1.26, 2.53)*	1.57	(0.96, 2.57)	2.18	(1.01, 3.29)*	0.77	(0.41, 1.46)
p for trend			0.002		0.136		<.001		0.185	
**UHg (μg/L)a**									
**Q1**	(0.10–0.23)	655		Reference		Reference		Reference		Reference
**Q2**	(0.24–0.35)	641	0.86	(0.63, 1.18)	0.96	(0.62, 1.48)	0.89	(0.55, 1.44)	0.79	(0.52, 1.21)
**Q3**	(0.36–0.64)	648	1.19	(0.86, 1.66)	0.93	(0.56, 1.55)	1.36	(0.88, 2.11)	1.32	(0.86, 2.03)
**Q4**	(0.65–8.70)	647	1.31	(0.88, 1.95)	0.83	(0.52, 1.33)	1.55	(0.98, 2.46)	1.37	(0.89, 2.11)
p for trend			0.101		0.466		0.022		0.043	

**UCd (µg/L)** [a]										
**Q1**	(0.05–0.22)	688		Reference		Reference		Reference		Reference
**Q2**	(0.22–0.45)	607	1.00	(0.70, 1.43)	0.79	(0.49, 1.27)	1.06	(0.67. 1.67)	0.84	(0.58, 1.21)
**Q3**	(0.45–0.87)	648	0.78	(0.56, 1.09)	1.13	(0.68, 1.88)	0.84	(0.53, 1.36)	0.61	(0.39, 0.95)
**Q4**	(0.87–16.82)	648	0.86	(0.59, 1.24)	0.85	(0.47, 1.56)	1.09	(0.67, 1.77)	0.85	(0.56, 1.29)
p for trend				0.218		0.794		0.838		0.264

[a]Total mercury

*p-value < 0.05

Abbreviations: CI, confidence interval, BPb, blood lead; BHg, blood mercury; UHg, urinary mercury; UCd, urinary cadmium; TC, total cholesterol; non-HDL, non-high density lipoprotein cholesterol; TG, triglyceride; LDL, low-density lipoprotein cholesterol

Covariates: age, gender, BMI, house income, education level, smoking states, drinking states, physical activity, and high seafood dietary group

Supplemental Material, Table S3. Heavy metals related occupation-adjusted OR (95% CIs) of serum lipid profiles by heavy metal concentrations in participants (n = 2,591)

		n	β (95% CI) of serum lipid levels							
			**Elevated TC** (≥200 mg/dL)		**Elevated TG** (≥150 mg/dL)		**Elevated LDL-C** (≥130 mg/dL)		**Elevated-Non_HDL-C** (≥160mg/dL)	
**BPb (μg/dL)**										
**Q1**	(0.37–1.21)	651		Reference		Reference		Reference		Reference
**Q2**	(1.21–1.65)	644	1.05	(0.76, 1.44)	1.04	(0.64, 1.48)	0.96	(0.60, 1.54)	1.03	(0.66, 1.60)
**Q3**	(1.65–2.23)	647	1.14	(0.87, 1.49)	0.88	(0.54, 1.43)	1.26	(0.85, 1.87)	1.28	(0.84, 1.93)
**Q4**	(2.23–20.58)	649	1.49	(1.07, 2.07)*	1.05	(0.61, 1.80)	1.58	(1.04, 2.42)*	1.71	(1.09, 2.68)*
p for trend			0.02		0.936		0.02		0.01	
**BHg (μg/L)[a]**										
Q1	(0.33–1.86)	647		Reference		Reference		Reference		Reference
Q2	(1.87–2.77)	648	1.17	(0.86, 1.59)	1.22	(0.75, 2.01)	1.63	(1.09, 2.46)*	1.03	(0.66, 1.60)
Q3	(2.77–4.30)	649	1.21	(0.87, 1.68)	0.94	(0.58, 1.52)	1.79	(1.18, 2.70)*	1.28	(0.84, 1.93)
Q4	(4.30–60.60)	647	1.72	(1.21, 2.44)*	1.40	(0.85, 2.31)	2.23	(1.51, 3.30)*	1.71	(1.09, 2.68)*
p for trend			0.004		0.312		<.001		0.01	
**UHg (μg/L) [a]**										
Q1	(0.10–0.23)	655		Reference		Reference		Reference		Reference
Q2	(0.24–0.35)	641	0.99	(0.65, 1.52)	0.87	(0.64, 1.19)	0.88	(0.54, 1.42)	0.79	(0.51, 1.20)
Q3	(0.36–0.64)	648	0.95	(0.57, 1.56)	1.19	(0.86, 1.66)	1.34	(0.86, 2.08)	1.31	(0.86, 2.01)
Q4	(0.65–8.70)	647	0.85	(0.53, 1.36)	1.31	(0.88, 1.96)	1.56	(0.98, 2.46)	1.37	(0.89, 2.10)
p for trend			0.492		0.104		0.021		0.041	

**UCd (μg/L)**									
Q1 (0.05–0.22)	688		Reference		Reference		Reference		Reference
Q2 (0.22–0.45)	607	1.00	(0.70, 1.42)	0.77	(0.47, 1.24)	1.08	(0.69. 1.71)	0.84	(0.58, 1.22)
Q3 (0.45-0.87)	648	0.77	(0.56, 1.08)	1.11	(0.67, 1.85)	0.87	(0.54, 1.39)	0.61	(0.39, 0.95)
Q4 (0.87-16.81)	648	0.85	(0.59, 1.22)	0.84	(0.46, 1.55)	1.13	(0.69, 1.84)	0.85	(0.56, 1.30)
p for trend			0.201		0.756		0.745		0.273

[a]Total mercury

*p-value < 0.05

Abbreviations: CI, confidence interval, BPb, blood lead; BHg, blood mercury; UHg, urinary mercury; UCd, urinary cadmium; TC, total cholesterol; non-HDL, non-high density lipoprotein cholesterol; TG, triglyceride; LDL, low-density lipoprotein cholesterol

Covariates: age, gender, BMI, house income, education level, smoking states, drinking states, physical activity, and heavy metals related-occupation group
